# Effects of *N*-glycans on the structure of human IgA2

**DOI:** 10.3389/fmolb.2024.1390659

**Published:** 2024-04-05

**Authors:** Valentina Ruocco, Clemens Grünwald-Gruber, Behzad Rad, Rupert Tscheliessnig, Michal Hammel, Richard Strasser

**Affiliations:** ^1^ Department of Applied Genetics and Cell Biology, University of Natural Resources and Life Sciences, Vienna, Austria; ^2^ Core Facility Mass Spectrometry, University of Natural Resources and Life Sciences, Vienna, Austria; ^3^ The Molecular Foundry, Lawrence Berkeley National Laboratory, Berkeley, CA, United States; ^4^ Division of Biophysics, Gottfried-Schatz-Research-Center, Medical University of Graz, Graz, Austria; ^5^ Molecular Biophysics and Integrated Bioimaging, Lawrence Berkeley National Laboratory, Berkeley, CA, United States

**Keywords:** *N*-linked glycan, IgA antibodies, SAXS, flexibility, protein assembly, protein stability

## Abstract

The transition of IgA antibodies into clinical development is crucial because they have the potential to create a new class of therapeutics with superior pathogen neutralization, cancer cell killing, and immunomodulation capacity compared to IgG. However, the biological role of IgA glycans in these processes needs to be better understood. This study provides a detailed biochemical, biophysical, and structural characterization of recombinant monomeric human IgA2, which varies in the amount/locations of attached glycans. Monomeric IgA2 antibodies were produced by removing the *N*-linked glycans in the CH1 and CH2 domains. The impact of glycans on oligomer formation, thermal stability, and receptor binding was evaluated. In addition, we performed a structural analysis of recombinant IgA2 in solution using Small Angle X-Ray Scattering (SAXS) to examine the effect of glycans on protein structure and flexibility. Our results indicate that the absence of glycans in the Fc tail region leads to higher-order aggregates. SAXS, combined with atomistic modeling, showed that the lack of glycans in the CH2 domain results in increased flexibility between the Fab and Fc domains and a different distribution of open and closed conformations in solution. When binding with the Fcα-receptor, the dissociation constant remains unaltered in the absence of glycans in the CH1 or CH2 domain, compared to the fully glycosylated protein. These results provide insights into *N*-glycans’ function on IgA2, which could have important implications for developing more effective IgA-based therapeutics in the future.

## 1 Introduction

In recent years, there has been a renewed interest in IgA antibodies. Studies have elucidated the secretory and dimeric IgA structure (Bharathkar et al., 2020), and recent evidence has shown the advantages of IgA in virus-neutralizing (Sterlin et al., 2021; Sun et al., 2021; Göritzer et al., 2024) and cancer-killing (Brandsma et al., 2019) which highlights the potential of IgA in immunotherapy. IgA is the most abundant immunoglobulin isotype produced in the human body, and contrary to IgG, it is present in monomeric and polymeric forms (de Sousa-Pereira and Woof, 2019). The polymeric form of IgA is primarily located at mucosal surfaces, serving as the first line of defense against pathogens. The monomeric form is mainly in the human blood serum, regulating anti-inflammatory and proinflammatory responses. The most extensively characterized cellular receptor of IgA is FcαRI, which is expressed in the blood by myeloid cells (Breedveld and Van Egmond, 2019). In humans, there are two IgA subclasses, IgA1 and IgA2. They differ in the length of the hinge region, IgA1 has a 13 amino acid residue extension absent in IgA2, as well as in the type and amount of glycosylation (Bakema and Van Egmond, 2011). Both subclasses are abundant in all organs and tissues except in the intestines, where IgA2 is predominant, and in the serum, where the IgA1 monomer is found at 90% (Davis et al., 2020). There are three allotypes of IgA2: IgA2 (m1), IgA2 (m2) and IgA2 (mn). The IgA (m2) and IgA2 (mn) allotypes form a canonical LC-HC disulfide bond, whereas, in IgA2 (m1), there is an LC-LC disulfide bond formation instead. Unlike IgG, IgA is a heavily glycosylated protein, containing two *N-*glycosylation sites and several *O-*glycosylation sites in IgA1, and four and five *N*-glycosylation sites in IgA2 (m1) and IgA2 (m2), respectively (Woof and Russell, 2011). Glycosylation affects the biological activity, solubility, protein conformation, serum half-life, and safety of therapeutic antibodies. Glycans play a crucial role in immunity, and aberrant glycosylation has been linked to the development of many diseases (Ding et al., 2022). Although IgG *N*-glycosylation has been extensively studied (Cobb, 2021), there is limited information available on IgA glycosylation and the biological function of distinct IgA glycans (Yoo et al., 2010; Steffen et al., 2020). This limited information on glycosylation is likely due to the structural complexity and the lack of a complete crystal structure (Pan et al., 2021). However, pharmacokinetic studies have demonstrated a clear correlation between the *N*-glycan sialylation and half-life in serum (Rouwendal et al., 2016). In IgG, the *N*-glycans are stabilizing intramolecular interactions between the two heavy chains and are restricted in flexibility (Remesh et al., 2018). In IgA, the *N*-glycans are located on the surface of the protein. However, no studies have yet assessed the flexibility of the *N*-glycans and their effect on the antibody backbone. The experimental validation of the Fc and Fab flexibility in solution has not been explored due to the absence of an accurate experimental technique that can track small motions in solution. This study used a glycoengineering approach to generate different IgA2 (m2) variants with mutated *N*-glycosylation sites. IgA2 (m2) was produced with all five native *N*-glycosylation sites intact and three mutants with removed Fab and Fc *N*-glycans. The expression of all glycoforms was assessed, along with the thermal stability and the thermodynamics of complex formation, using a range of biochemical and biophysical methods. Using small-angle X-ray scattering (SAXS), we characterized the structure of the different glycoforms in solution to investigate the impact of the removal of the glycans on the conformation. The experimental SAXS data combined with the modeling of the full-length antibody allowed us to detect the flexibility of individual Fab domains, providing experimental validation of increased Fab flexibility in solution in correlation with the absence of *N*-glycans.

## 2 Results

### 2.1 Production of different IgA2 (m2) glycosylation variants

**FIGURE 1:**
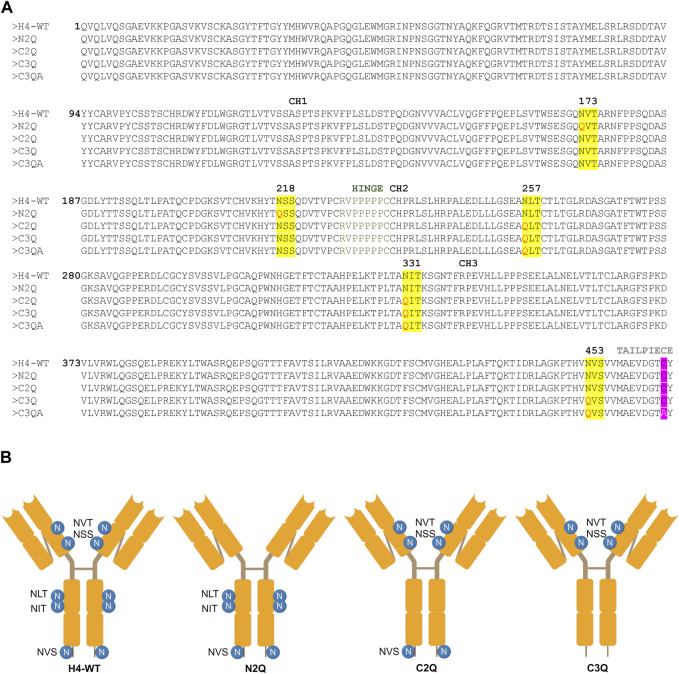
Amino acid sequence alignment and depiction of IgA2 (m2) variants. **(A)** Alignment of protein sequences of constant regions for IgA2 (m2) H4-WT and the generated mutants. *N*-linked glycosylation sites are highlighted in yellow, and exchanged amino acid residues are highlighted in red. The penultimate cysteine is highlighted in pink, and the alanine in the C3QA variant is shown in white. **(B)** Schematic illustration of the assembled IgA2 (m2) variants with *N*-glycosylation sites indicated by the specific sequon.

**FIGURE 2:**
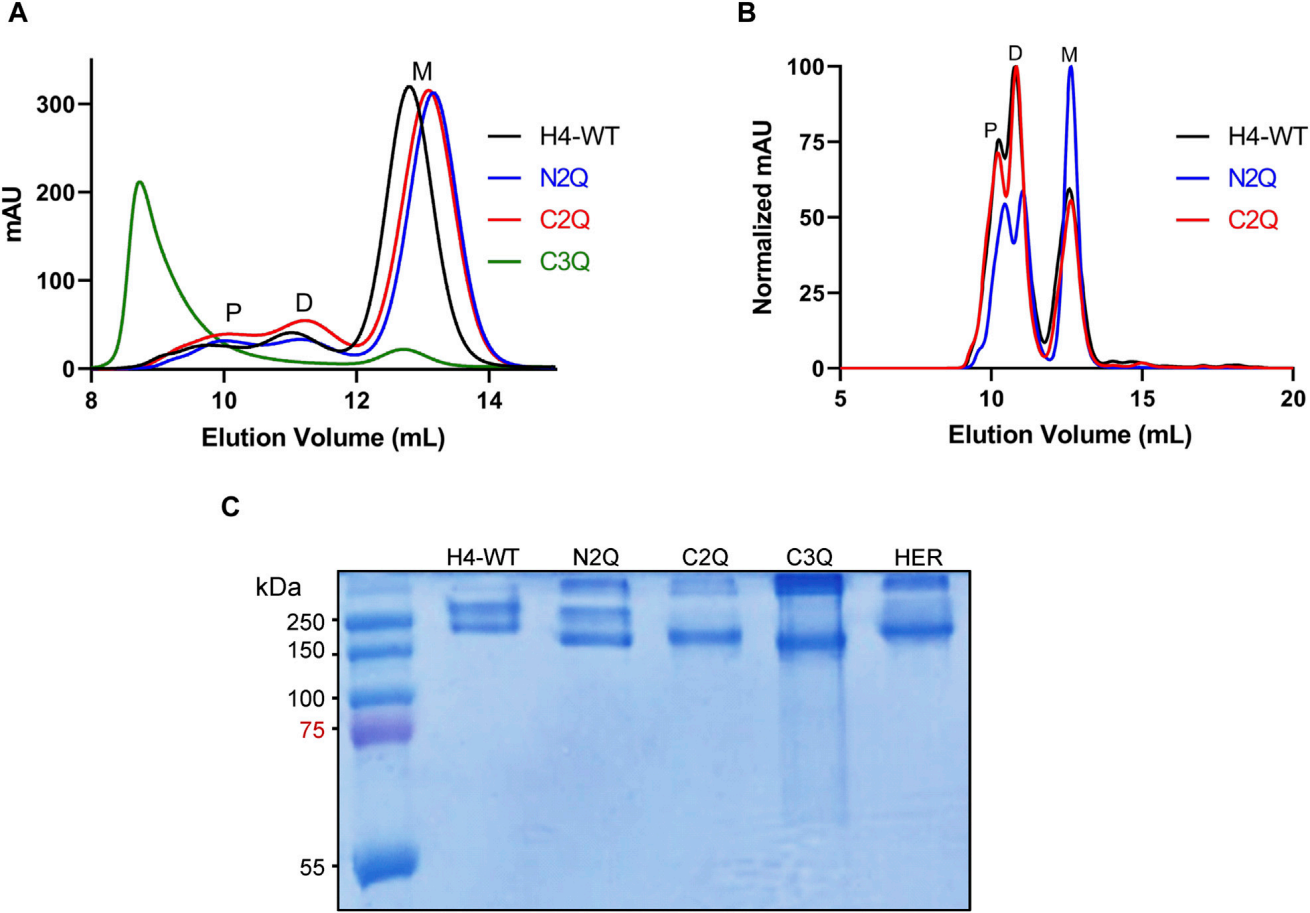
Purification and analysis of the IgA (m2) assembly. **(A)** Overlay of analytical size-exclusion chromatograms of affinity-purified monomeric IgA2 (m2) from small-scale transient transfections. Monomer (M), dimer (D), and polymer (P) peaks are indicated. No JC was co-expressed. **(B)** Overlay of normalized analytical size-exclusion chromatograms of affinity-purified dimeric IgA2 (m2) from small-scale transient transfections. JC was co-expressed to increase the amounts of dimeric IgA2 (m2). **(C)** SDS-PAGE under non-reducing conditions of purified monomeric IgA2 (m2) glycosylation mutants followed by Coomassie Brilliant Blue staining. Herceptin IgA2 (m2) (HER) is included for comparison.

To assess the impact of *N*-glycosylation on IgA2 (m2) production, we produced several mutants that lacked distinct *N*-glycosylation sites. We produced a WT variant of the SARS-CoV-2 H4 antibody (Wu et al., 2020) with all five native *N*-glycosylation sites, the C2Q mutant in which we removed the glycosylation sites NLT and NIT in the CH2 domain, the N2Q mutant in which we removed the glycosylation sites NVT and NSS in the CH1 domain and the C3Q in which we removed the *N*-glycosylation site in the tailpiece in addition to the CH2 *N*-glycans (NLT, NIT, NVS) (Figure 1). In addition, a C3Q variant (C3QA) was generated by replacing the penultimate cysteine with alanine. The transient expression of IgA2 (m2) WT, N2Q, and C2Q resulted in similar quantities after CaptureSelect affinity purification, while C3Q and C3QA exhibited poor yields. Size-exclusion chromatography purification revealed that WT and mutants N2Q and C2Q displayed a primary peak corresponding to the monomer and smaller peaks corresponding to the dimer and larger oligomeric species (Figure 2A). The peak for the WT monomer displayed a slight shift and eluted earlier. This is likely due to the higher molecular weight resulting from the presence of two fully *N*-glycosylated heavy chains. The lack of two *N*-glycans in the N2Q and C2Q mutants did not affect oligomer formation, as the ratio between monomers, dimers, and polymers did not significantly change across different batches. However, the glycosylation variant C3Q showed a small peak for monomers and a more prominent peak for high molecular weight oligomers (Figure 2A).

The absence of the *N*-glycan in the tailpiece is known to cause the formation of larger oligomers (Xie et al., 2021; Pan et al., 2024). Lombana et al. (2019) previously observed this phenomenon during dimeric IgA2 (m2) production when co-expressing heavy chain (HC), light chain (LC), and the joining chain (JC). Here, we observed that this aggregation occurs without the JC (Figure 2A). We hypothesize that the *N*-glycan in the NVS site shields the penultimate cysteine, thereby preventing disulfide bridge formation. The NVS site may be underglycosylated to some degree, which could correlate with the presence of a small amount of dimer, even in the absence of the JC (Figure 2A). To determine whether the lack of three *N*-glycans in C3Q affects overall hydrophobicity and, consequently, the propensity to aggregate, a C3Q variant with an alanine instead of the penultimate cysteine was generated. The new variant, C3QA, exhibited significantly fewer high molecular weight oligomers compared to C3Q, confirming the hypothesis that the penultimate cysteine is being shielded by the *N*-glycan (Supplementary Figure S1). The expression of IgA2 (m2) WT, N2Q, and C2Q was tested in their dimeric form by co-expressing the LC, HC, and JC in a proportion 1:1:0.5. The C3Q variant was not included due to poor expression. The yield of the three variants was comparable. The predominant peak for H4-WT and C2Q was the one corresponding to dimers, while the N2Q glycosylation variant still showed a significant amount of monomer (Figure 2B). Overall, the assembly is maintained in forming the dimer, and the SEC profile resembles the previously published data (Lombana et al., 2019), indicating a minor role of the CH1 and CH2 *N*-glycans in the dimeric assembly. Isolated aliquots of the monomeric form of IgA2 (m2) were utilized for all subsequent analyses (Figure 2C).

### 2.2 The impact of removing distinct *N*-glycans on the composition of the remaining IgA2 (m2) *N*-glycans

The composition and structure of IgA *N*-glycans are considerably heterogeneous in individuals and different sources (serum or recombinant) (Gomes et al., 2008). Several *N*-glycan structures have been identified on the five *N*-glycosylation sites on the IgA2 HC (Ding et al., 2022). Unlike IgG glycans, which are oriented towards the interior of both CH2 domains, molecular modeling revealed that IgA *N*-glycans were oriented towards the exterior of the molecule (Mattu et al., 1998). The external orientation of IgA *N*-glycans makes them more accessible to the glycosyltransferases in host cells, resulting in cell-specific glycoforms and extensive *N*-glycan heterogeneity (Mattu et al., 1998). To investigate the type of *N*-glycan attached to each site of monomeric IgA2, we performed site-specific analysis using LC-MS/MS. The analysis aimed to evaluate whether removing glycans from a specific site influences the overall N-glycosylation profile on other sites, which would hint at conformational changes.

**FIGURE 3:**
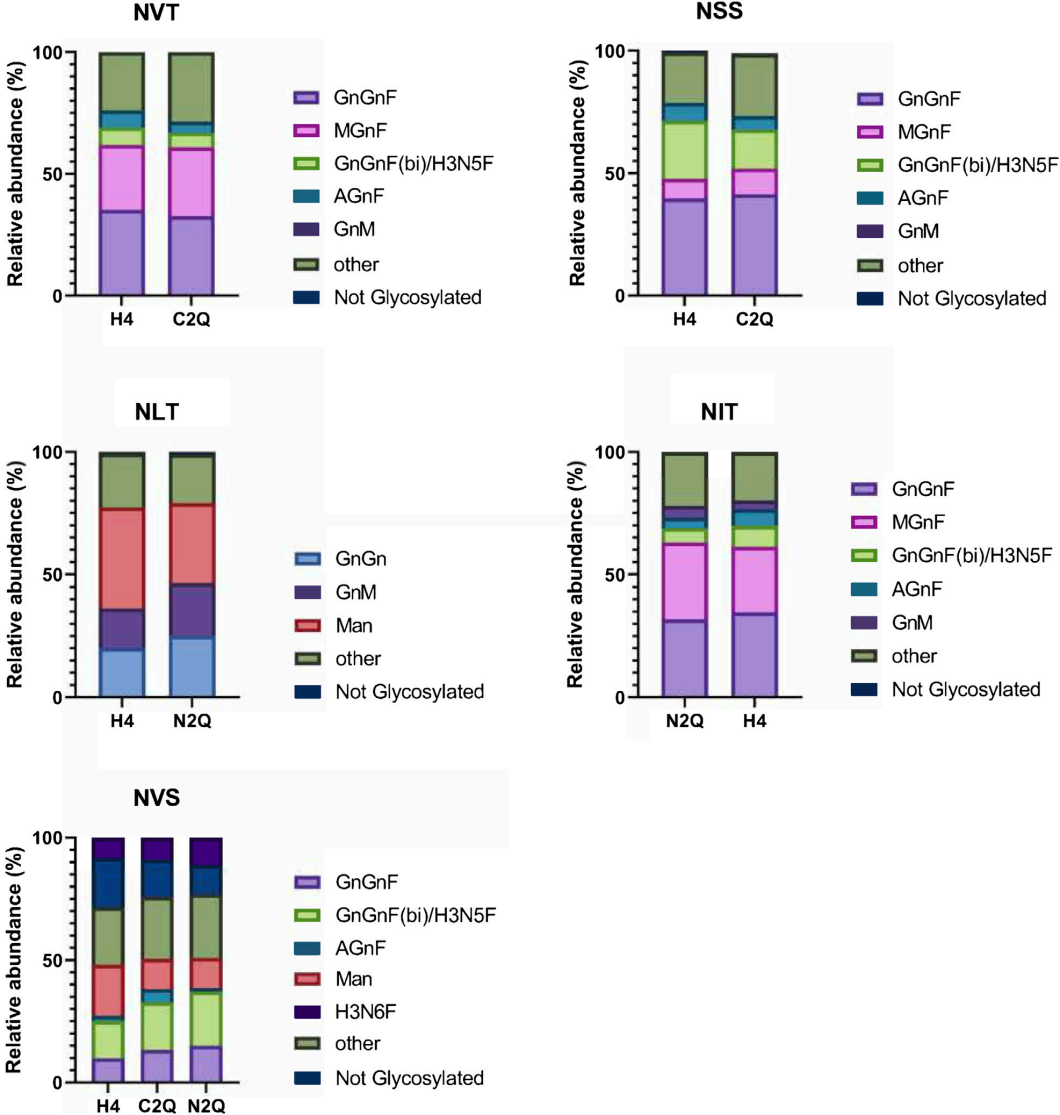
Site-specific analysis of *N*-linked glycans on the different IgA2 (m2) variants. The visual representation highlights the more abundant species while grouping those making up less than 4% into an “other” category for clarity. The *N*-glycosylation sites are presented in order from N- to C-terminus.

In Figure 3, the relative abundance of the most abundant *N*-glycans is shown for each *N*-glycosylation site. Overall, the *N*-glycan profiles are quite heterogeneous, with apparent site-specific differences. The *N*-glycan structures in IgA2 (m2) WT mostly consist of biantennary fucosylated complex *N*-glycans. WT and N2Q have a higher level of oligomannosidic glycans at the NLT site (41% and 32%, respectively), and the complex *N*-glycans at this site lack fucose residues. The glycosylation site at the tailpiece, NVS, is not glycosylated to a certain extent, with levels of 20% for WT, 15% for C2Q and 11% for N2Q (Figure 3). The *N*-glycans at the NSS and NVS sites display more multibranched structures. All variants are site-specifically modified at the NVT, NSS, and NIT sites, with similar trimming/maturation. These branching structures suggest that the local environments surrounding these asparagine residues are similar and that the absence of individual *N*-glycans has little impact on *N*-glycan processing.

### 2.3 The impact of distinct *N*-glycans on IgA2 (m2) thermostability

**FIGURE 4:**
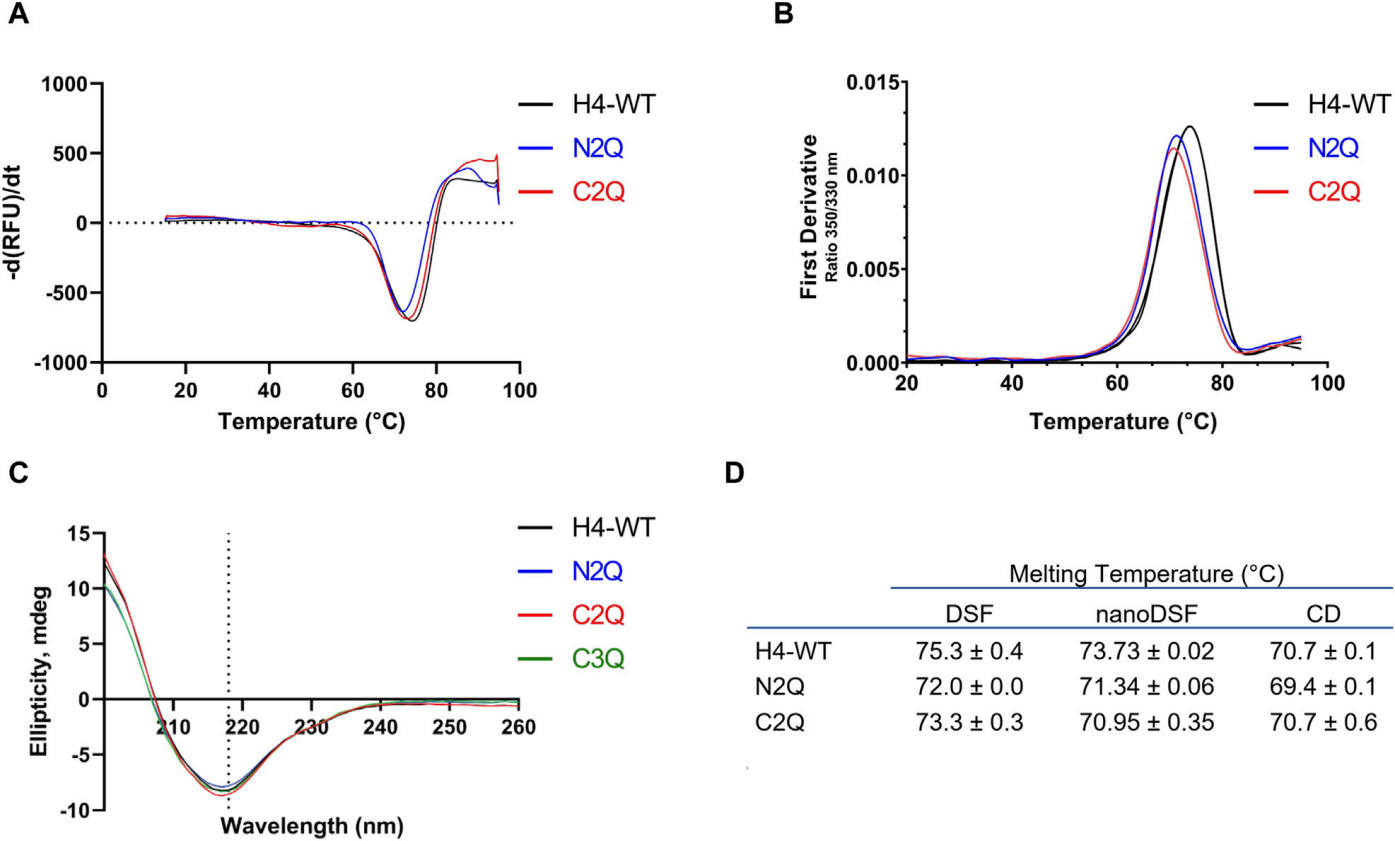
Thermostability and secondary structure analysis of the different IgA2 (m2) variants. **(A)** Melting curves of IgA2 (m2) variants obtained by DSF. **(B)** The thermal unfolding transition was monitored using IgA2 intrinsic tryptophan fluorescence by nanoDSF. **(C)** CD spectra of IgA2 (m2) variants. Representative images of single measurements are shown. **(D)** Summary of the melting temperatures measured by DSF, nanoDSF, and CD. All experiments were repeated three times (*n* = 3).

To assess the impact of *N*-glycosylation on the thermostability of the different glycoforms, we employed three different methods: differential scanning fluorimetry (DSF), nanoDSF, and CD. As previously shown (Lombana et al., 2019), only one melting transition was observed. All three methods determined the melting temperature (T_m_) and indicated that the IgA2 (m2) WT, containing all five *N*-glycans, is approximately 2°C more stable than the other glycoforms (Figures 4A, B). The results suggest that the *N*-glycans contribute to thermal stability. However, unlike a previous study (Pan et al., 2024) which reported a variation of 4°C upon removal of two glycans in IgA1, the differences in T_m_ are less pronounced. CD spectroscopy experiments were carried out to investigate further the effect of different *N*-glycosylation mutants on the IgA2 (m2) conformation. Far-UV spectra between 260 and 200 nm were recorded for the different variants. The IgA2 (m2) CD spectra showed a minimum at 218 nm, indicating a predominance of β-sheets and a low abundance of α-helical structures (Figure 4C; Supplementary Figure S2). This CD spectrum is consistent with the typical antibody structure and suggests that removing *N*-glycans does not alter the secondary structure of IgA2 (m2) (Figure 4D).

### 2.4 The impact of distinct *N*-glycans on IgA2 (m2)-FcαRI binding

**FIGURE 5:**
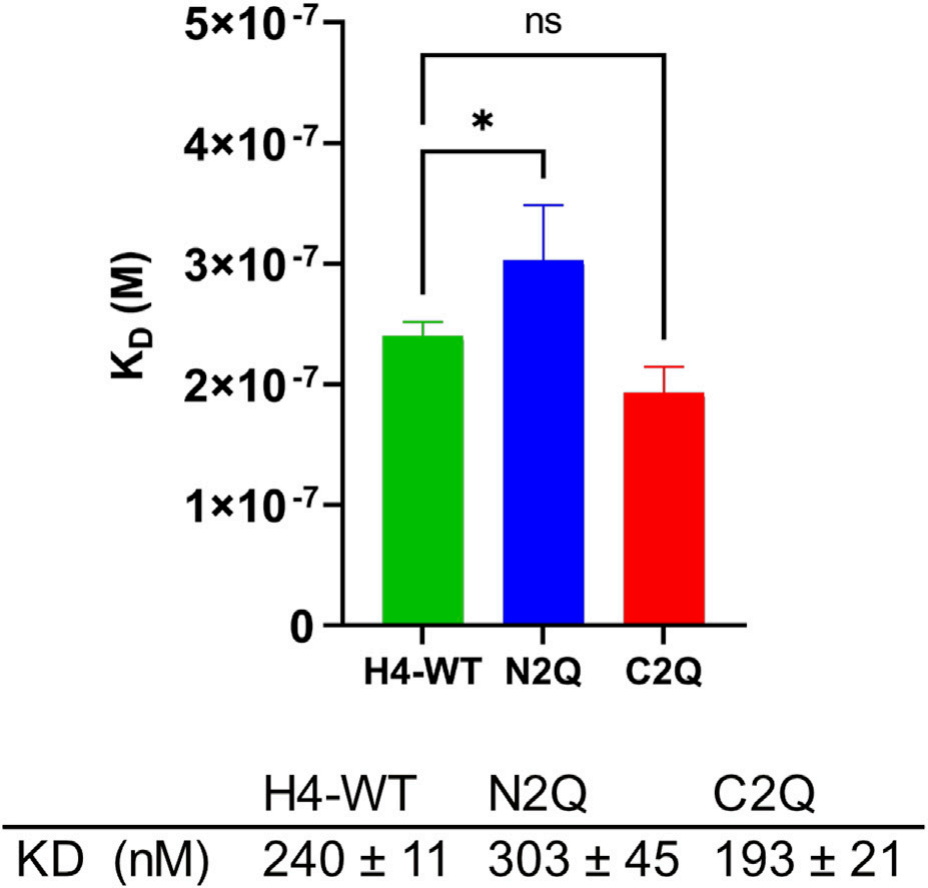
Binding affinity of IgA2 (m2) glycosylation variants to the FcαRI receptor. All experiments were repeated three times, and mean values ± SD are shown. Significance levels are shown according to a Student’s *t*-test; ns, not significant, “*” *p* < 0.05.

Recent research suggests that unbound serum IgA, which lacks an antigen, can modulate immune responses through inhibitory signaling involving the immunoreceptor tyrosine-based activation motif (ITAM) (Davis et al., 2020). This modulation promotes anti-inflammatory responses, which are crucial for maintaining the stability of the human body’s internal environment (Ding et al., 2022). Monomeric IgA binds to FcαRI via its CH2 and CH3 domains. The difference in the binding mode between FcαRI and IgA1 and IgA2 has not been extensively studied. However, it is known that IgA1 has a lower K_D_ compared to IgA2 (Göritzer et al., 2019). Additionally, it has been shown that distinct FcαRI *N*-glycans modulate the binding affinity to IgAs (Göritzer et al., 2019). However, less is known about the role of specific IgA2 *N*-glycans in binding with the FcαRI receptor (Carayannopoulos et al., 1994; Oortwijn et al., 2007). To investigate the impact of distinct *N*-glycans on the binding of IgA2 (m2) to FcαRI, SPR spectroscopy of the *N*-glycosylation mutants of IgA2 (m2) and FcαRI was conducted. Despite the crystal structure of the IgA1–FcαRI complex suggesting a 1:2 stoichiometry (Herr et al., 2003; Posgai et al., 2018), the response units obtained from the binding curves in SPR experiments indicated a 1:1 binding model. Therefore, the sensorgrams were fitted accordingly. The IgA2 glycoforms exhibit an equilibrium dissociation constant in the 200–400 nM range, consistent with previous studies (Göritzer et al., 2019; Lombana et al., 2019). The glycoforms have similar K_D_ values, with N2Q showing a slightly higher K_D_, which suggests a small allosteric effect resulting from the removal of Fab glycans on the binding site of FcαRI (Figure 5; Supplementary Figure S3).

### 2.5 Structural characterization IgA2 (m2) *N*-glycosylation variants with SAXS

**FIGURE 6:**
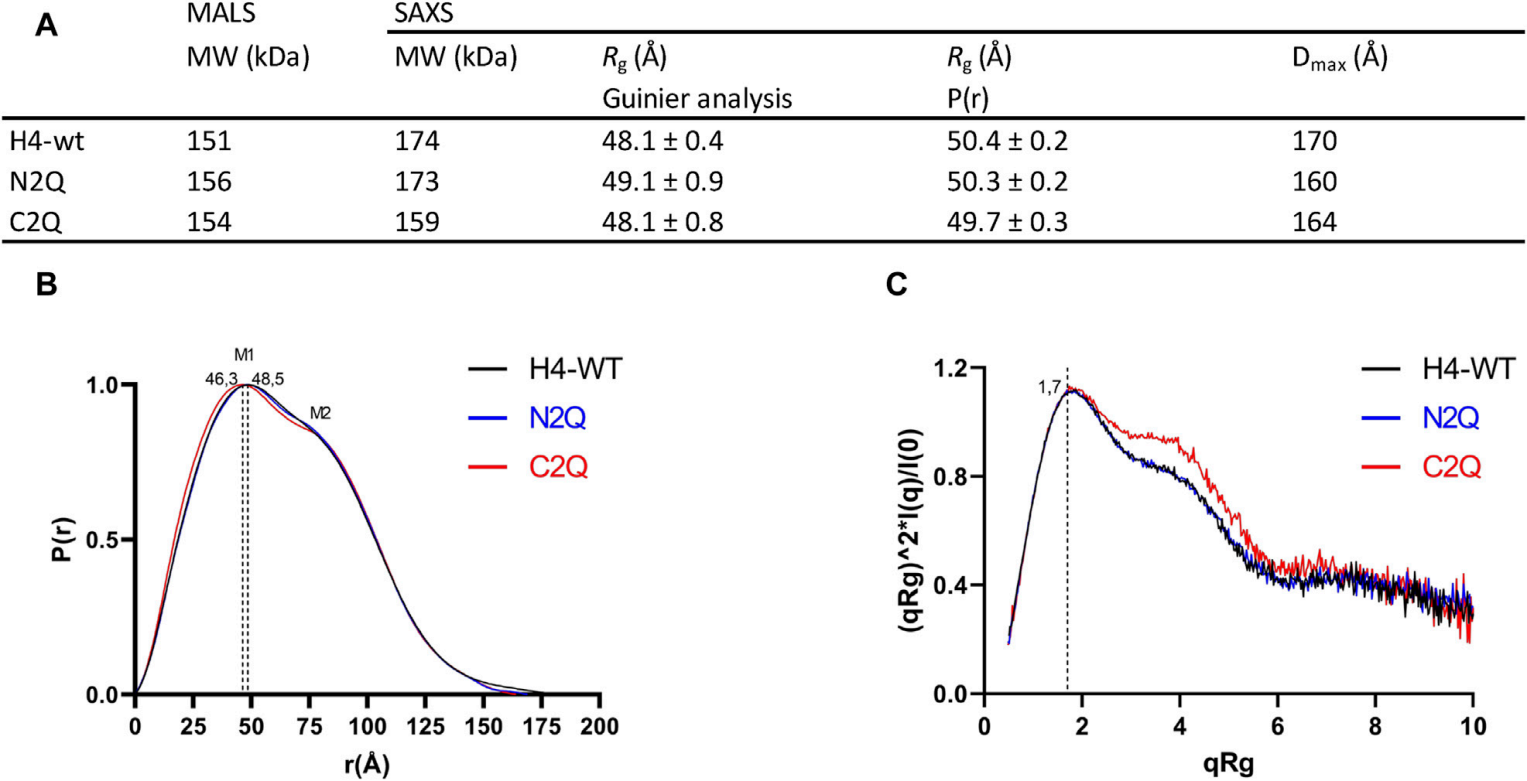
Experimental SAXS profiles. **(A)** Table of SAXS and MALS experimental parameters. **(B)** P(r) functions calculated for the experimental SAXS curves for all tested glycoforms (colored as indicated). The P(r) functions are normalized at the maxima. The P(r) shoulder at r ∼ 80 Å indicates the Fab-Fc separation described within the atomic model of IgA2. The P(r) peak at 40 Å corresponds to the average size across Fc or Fab regions. **(C)** Normalized Kratky plot.

Small angle X-ray scattering (SAXS) was used to characterize the IgA2 (m2) flexibility of all three glycoforms in solution. The data quality was assessed by evaluating the Guinier plot to ensure accurate structural interpretations. Guinier analyses produced linear plots for all three experimental SAXS profiles (Supplementary Figure S4). The *R*_g_ values for all samples were similar, within the range of 48–49 Å (Figure 6A). The IgA2 (m2) *R*_g_ value here differs from the previously reported value (Furtado et al., 2004). We reported smaller *R*_g_ values, most likely because we performed a size exclusion chromatography coupled SAXS (SEC-SAXS) that eliminated non-specific oligomerization or aggregations in the sample. The maximal dimensions (Dmax) of the IgA2 (m2) variants were determined by the pair distribution function P(r). The maximal dimensions were approximately ∼170 Å for H4-WT and ∼160 Å for C2Q and N2Q. In the P(r), M1 and M2 maxima correspond to the structure’s most frequently occurring interatomic distance. The first P(r) function peak at r ∼ 45 Å arises from the approximate repeated distances across the Fc or Fab regions’ length and breadth (Hodge et al., 2021). The P(r) shoulder at r ∼ 80 Å reflects the inter-domain distances between the Fc and Fab regions (Figure 6B). The difference between the P(r) peak and shoulder indicates the Fc-Fabs’ distancing, which correlates with the extended conformers’ occupancy in solution. The P(r) shapes of the different IgA2 (m2) were similar but distinct. The peaks in H4-WT and N2Q are less separated, indicating a more compact shape for these variants. Conversely, the curve of C2Q is shifted to the left, and the peaks M1 and M2 are more separated. This correlates with a lack of CH2 *N*-glycans, which could translate to smaller interatomic distances in the Fc region and a shift in the M1 peak.

The Kratky plot analysis of SAXS data is an effective method to evaluate the compactness of a protein and the presence of disordered regions (Hammel, 2012). The bell-shaped Kratky plot becomes asymmetrically stretched for a multidomain IgA2 protein connected by linkers but still adopts a folded character, as shown by the position of maxima at q*R*_g_ = 1.7 (Figure 6C). The dimensionless Kratky plot, where I (q) is normalized by I (0) and q is normalized by *R*_g_, allows for the comparison of proteins of different sizes (Hammel, 2012). The plot displays curves typical of a multidomain protein with domains connected by flexible linkers. Shoulders at q*R*_g_ ∼ 4 in the C2Q variant indicate a more distinct separation between Fc and Fab domains than in the H4-WT and N2Q cases (Figure 6C). In summary, it has been observed that all the glycoforms show a typical antibody structure in solution. Additionally, experimental SAXS curves indicate that the C2Q variant, which lacks CH2 *N*-glycans, displays altered mobility of the Fab domains compared to WT and N2Q.

### 2.6 Visualizing of IgA2 (m2) mobility by SAXS modeling

**FIGURE 7:**
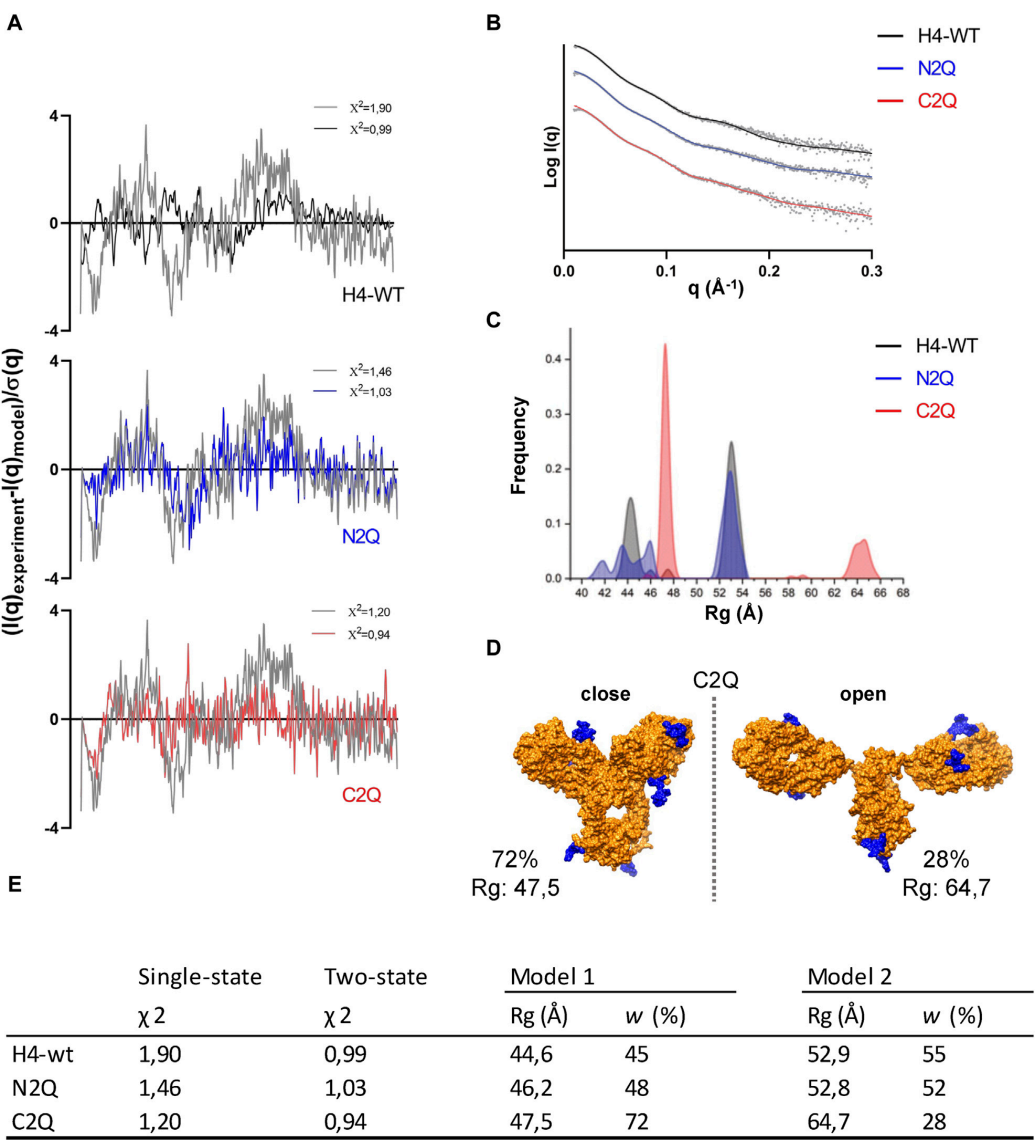
The flexibility of IgA2 (m2) glycosylation variants. **(A)** Residuals (experiment/model) for the fits of the two-state models (black, blue, and red) are presented alongside the optimal single model in grey. These residuals collectively indicate the necessity of employing the two-state model to fit the experimental SAXS curves satisfactorily. **(B)** The panel displays experimental SAXS profiles of the IgA2 glycoforms (scatter) with the corresponding theoretical SAXS profiles derived from their respective two-state atomistic models (line). **(C)** Histograms of the *R*_g_ distributions of the top 300 selected multistate models are shown for the two-state model. **(D)** Representation of the open and closed two-state models for C2Q. The glycan moiety is colored blue. **(E)** Modeling parameters *R*_g_ and w of IgA2 (m2) variants. Results from the best-fitting multistate models are shown.

As the crystal structure of the full-length IgA2 (m2) antibody has not been determined, and the only deposited structure (1R70) was obtained by solution scattering, atomistic models obtained with machine learning algorithms were used to enhance the interpretation of SAXS data. The amino acid sequence of the antibody was submitted to AlphaFold-Multimer to obtain the predicted structure (Jumper et al., 2021). In addition to the predicted structure in PDB format, the database also provides a predicted align error (PAE) (Supplementary Figure S5). With the development of accurate structure prediction algorithms such as AlphaFold or RosettaFold (Baek et al., 2021) validating the predicted structure with SAXS is becoming increasingly popular (Chinnam et al., 2022). As noted in previous studies (Hodge et al., 2021), the single conformation predicted by AlphaFold does not match the SAXS data. However, it serves as a starting point for generating an ensemble of conformations. To explore the conformational space of the Fab regions relative to the Fc regions, we used the BILBOMD program (Pelikan et al., 2009). This conformational sampling resulted in a pool of over 10.000 atomistic models, from which SAXS curves were calculated (Schneidman-Duhovny et al., 2013) and compared to the experimental curve. MultiFoXS (Schneidman-Duhovny et al., 2016) provides the weighting of multistate models that fit the experimental data better than a single best-fit model. Two distinct conformations, open and closed, are required to achieve a certain level of accuracy in fitting the SAXS data. The goodness of fit measured by χ2 quantifies the experimental and theoretical differences (Figures 7A, B).

The fit of the SAXS data was significantly improved using a two-state model for each of the three variants. For example, as seen in Figure 7A, the χ2 value of the one-state model of H4-WT decreases from 1.90 to 0.99 with the two-state model. However, the three-state model did not improve the SAXS fit for the three variants. To estimate the number of conformational states in the solution, we examined the *R*_g_ distribution for the top 300 selected multistate models (Schneidman-Duhovny and Hammel, 2018). The distribution of *R*_g_ in the two-state models (Figure 7C) displays two primary peaks for H4-WT and N2Q. One peak corresponds to closed conformations at *R*_g_ 43–46 Å, while the other corresponds to open conformations at *R*_g_ 52–54 Å. For C2Q, the closed conformation is described by a sharp peak around 47 Å, while the open conformation is much larger, at 63–66 Å (Figures 7D, E). The *R*_g_-distribution curve indicates a similar population of the closed and open conformers for H4-WT and N2Q. However, the shift in peaks in C2Q suggests less population of the open conformation (Supplementary Video).

Our findings demonstrate that an ensemble of conformations should describe an antibody’s conformation. Additionally, we observed that removing the CH2 glycans from IgA2 (m2) resulted in the acquisition of different conformations compared to WT and N2Q. Specifically, the flexibility of the C2Q Fabs increased, leading to a more open shape.

## 3 Discussion

There has been a significant increase in interest in using IgA immunoglobulins for therapeutic purposes in recent years. This interest is due to the demonstrated effectiveness of the IgA subclass, particularly in eradicating tumors and in the emerging field of mucosal immune therapy (Sterlin and Gorochov, 2021). Utilizing the IgA-specific receptor FcαRI on myeloid cells, IgA antibodies exhibit superior tumour-killing capabilities compared to IgG (Brandsma et al., 2019). Although there are promising aspects, the introduction of IgA into clinical practice faces challenges due to its intrinsic properties. These include the complexities of production and purification, a relatively short serum half-life, and a highly variable glycosylation profile. Additionally, assessing IgA efficacy in pre-clinical studies is complicated as mice lack natural expression of FcαRI. In this study, we developed a new approach for relating the structure to stability and function of IgA2 antibodies, assessing the effects of distinct *N*-glycans that are still debated.

Several studies have shown that the glycan profile can have a significant impact on protein function (Jennewein and Alter, 2017; Schjoldager et al., 2020). However, in this study, we found that removing glycans from either the Fc or Fab domain only caused a minor change in thermal stability and assembly. It is worth noting that the tailpiece glycan plays a key role in the assembly process by interacting with the tailpiece cysteine and acting as a shield. Removing the *N*-glycan from the Fab or Fc region had no significant effect on the dimer-to-monomer ratio, regardless of the absence or presence of the J chain. In contrast to the study of Lohse et al. (2016), which reported a change in the global *N*-glycan profile upon removal of the NIT glycosylation site from the CH2 domain of IgA2, the *N*-glycan profiles of our IgA (m2) mutants are very similar. This discrepancy may arise from other mutations included in the IgA2, as noted in their study.

In line with previous data, *N*-glycans do not play a direct role in FcαRI binding, as demonstrated by similar K_D_ values for different variants. However, it would be interesting to explore *N*-glycans’ role in binding with other receptors, such as the asialoglycoprotein receptor, DC-SIGN, or other lectin-type receptors. There are still open questions regarding the interaction and binding among the different IgA subtypes, while the structures’ differences are more elucidated. Most studies have focused on IgA1, the most abundant IgA subtype, with less attention given to heavily glycosylated IgA2. Structural studies of IgAs have recently advanced (Pan et al., 2024), and the solution structure of secretory IgA has been resolved by electron microscopy (Bharathkar et al., 2020).

Additionally, the structure of IgA1 in a complex with a specific bacterial protease has been reported (Wang et al., 2020). However, the resolution of the monomeric full form of IgA remains challenging due to the molecule’s intrinsic flexibility. Methods that rely on grids or crystals, such as electron microscopy, often fail to capture the dynamic nature of antibody conformations in a solution (Jay et al., 2018). The first structure of human IgA2 was obtained by Furtado et al. using X-ray and neutron scattering (Furtado et al., 2004). SEC, coupled with SAXS, has allowed for a more sophisticated analysis of the protein’s flexibility in solution. The starting model of the antibody is crucial for fitting the experimental SAXS curves. The IgA2 (m2) model predicted with AlphaFold-Multimer demonstrated accurate backbone prediction but less confidence in the hinge region and tailpiece of the antibody. This report presents the first solution structure of IgA2 (m2) obtained with SEC-SAXS. The results indicate that two conformations, close and open, better represent the antibody conformation in solution. To account for the system’s flexibility, an ensemble of models was selected to describe best the SAXS data from a large number of generated models.

The IgA2 (m2) C2Q variant, lacking Fc glycans, exhibits greater flexibility than H4-WT and N2Q variants. It is also more likely to adopt an extended structure, with *R*_g_ = 64,7 Å, even without a long hinge region. This may be attributed to long-distance interactions between the Fab and the CH2 *N*-glycans that keep the conformation close in WT IgA2 (m2). Non-covalent interactions between the Fab and Fc regions have also been observed in IgG1 antibodies. These findings demonstrate the complex allosteric interactions that govern antibody dynamics and asymmetry (Natesan and Agrawal, 2022). The used approach is effective in modeling the intrinsic flexibility of antibodies and has been applied to characterize other types of immunoglobulins (Hodge et al., 2021; Belviso et al., 2022). On the other hand, a disadvantage is describing each domain of the antibody (Fab and Fc) as a rigid unit. We added flexibility to the glycans modelled on the structures to circumvent this limitation. A similar approach was used to investigate the dynamics of IgA *N*-glycans through NMR (Oortwijn et al., 2007). It is known that the flexibility of molecules, rather than just the specific amino acids they are composed of, plays a crucial role in protein interactions (Teague, 2003). Antibodies with greater flexibility may adopt a wider range of conformations, enabling them to bind to a broader range of antigens (Ovchinnikov et al., 2018). Modulating antibody flexibility through glycosylation engineering or introducing backbone mutations is currently an understudied field in antibody engineering, which has great potential for the development of broadly binding antibodies. Understanding the structural features of glycosylated IgA2 (m2) in the solution can aid in designing recombinant IgA2 variants that are optimized for interaction with different cellular receptors or diverse antigens, making them more potent as therapeutics.

## 4 Materials and methods

### 4.1 Construct design and cloning

The codon-optimized genes of the heavy chains and light chain required for expression of the different IgA2 (m2) glycosylation variants in HEK293-6E cells were synthesized by GeneArt (Thermo Fisher Scientific, United States). The sequence of the variable region heavy chains (α-HC) and kappa light chain (κ-LC) were taken from the sequence of IgG-H4 (Kallolimath et al., 2021) and fused to the constant region of human IgA2 (m2). Sequences of the HCs and the κ-LC used for the expression in HEK293-6E were flanked with the coding sequence for the signal peptides “MELGLSWIFLLAILKGVQC” and “MDMRVPAQLLGLLLLWLSGARC,” respectively, and the restriction sites *Xba*I and *Bam*HI. The synthesized DNA was amplified by PCR with the primers “Strings_9F (CTTCCG- GCTCGTTTGTCTAGA)/Strings_2R (AAA​AAC​CCT​GGC​GGG​ATC​C).” The corresponding genes for the α-HCs and the κ-LC were then separately cloned into the *Xba*I/*Bam*HI sites of the mammalian expression vector pTT5 (National Research Council of Canada). The codon optimized gene for expression of the joining chain (JC) (AK312014.1) in HEK293-6E cells was synthesized by GeneArt (Thermo Fisher Scientific, United States) and included the coding sequence for the signal peptide “MELGLSWIFLLAILKGVQC” and the restriction sites *Bam*HI/*Sal*I used for cloning. The sequence of the glycosylation variant N2Q was synthesized with the mutation N to Q in the glycosylation sites NVT and NSS, the glycosylation variant C2Q with the mutation N to Q in the glycosylation sites NLT and NIT, the glycosylation variant C3Q with the mutation N to Q in the glycosylation sites NLT and NIT and NVS (Figure 1). The cloning and expression of the extracellular domain of the human FcαRI (P24071.1) was performed as described previously (Göritzer et al., 2019).

### 4.2 Recombinant production of IgA2 variants and FcαRI in HEK293-6E cells

The HEK293-6E cell line that constitutively expresses the Epstein−Barr virus nuclear antigen 1 of the Epstein−Barr virus was licensed from the National Research Council (NRC) of Canada (Durocher et al., 2002). The suspension cells were cultivated and transfected according to the manufacturer’s manual in F17 medium supplemented with 0.1% Pluronic F-68, 4 mM L-glutamine (Life Technologies, Germany), and 50 mg/L G418 (Biochrom, Germany). The cells were maintained in shaker flasks at 37°C in a humidified atmosphere with 5% CO_2_ on an orbital shaker never exceeding a cell density of 2×10^6^ cells/mL. For transient transfection of a 200 mL culture, cells were brought to a concentration of 1.7 × 10^6^ cells/mL. High-quality plasmid preparations of the pTT5 vector coding for the κ-LC and the different α-HCs were obtained using the PureYield Plasmid Midiprep System (Promega, United States). A total of 200 μg plasmid-DNA, consisting of 100 μg LC and 100 μg of the respective HC, were mixed with 10 mL of fresh medium. Another 10 mL of fresh medium, containing 2.5 μg/mL linear polyethylenimine (PEI) (Polysciences, Germany), was added to the DNA solution and incubated for 10 min. After adding the DNA/PEI mixture, the cells were incubated for 48 h, supplemented with 0.5% (w/v) tryptone N1 (Organotechnie, France) and further cultivated for 72 h. Supernatant containing the secreted soluble protein was harvested by centrifugation at 25,000 g for 30 min at 4°C and additionally filtrated (0.45 μm Durapore membrane filter, Merck Millipore, Germany). For the transient expression of monomeric and dimeric IgA2 in HEK293-6E cells, cultures were transfected with the κ-LC, the different α-HCs and JC constructs in a 1:1:0 and 1:1:0.5 ratio of µg DNA, respectively, as described in (Göritzer et al., 2019).

### 4.3 Purification of recombinant IgA2 (m2) and FcαRI

The supernatant of HEK293-6E suspension cells were subjected to a peristaltic pump packed with 1 mL of CaptureSelect IgA affinity resin (Thermo Fisher Scientific, United States) equilibrated with phosphate-buffered saline (PBS) pH 7.4. Proteins were eluted with 0.1 M glycine pH 2.8, followed by immediate addition of 6 μL of 2 M Tris pH 12 to each 1 mL fraction to neutralize the acidic pH from glycine elution. Highly concentrated fractions were pooled and dialyzed against PBS at 4°C overnight using SnakeSkin Dialysis Tubing with a MWCO of 10 kDa (Thermo Fisher Scientific, United States). Finally, the column was regenerated with 0.1 M glycine pH 2.5 and washed with PBS. Pooled protein fractions were then further concentrated using Amicon centrifugal filters with a MWCO of 10 kDa (Merck Millipore, Germany) and subjected to size-exclusion chromatography (SEC) on a HiLoad 16/600 Superdex 200 pg column (Cytiva, United States) equilibrated with PBS supplemented with 200 mM NaCl. For the recombinant production of the extracellular domain of the FcαRI, the cell culture supernatants were harvested after 6 days and diluted 1:2 in loading buffer (20 mM Tris-HCl pH 7.4, 500 mM NaCl, and 10 mM imidazole). The solution was loaded onto a 5 mL HisTrap HP column (Cytiva) equilibrated with 5 column volumes of loading buffer, and bound protein was eluted by applying 250 mM imidazole. Eluted fractions containing the protein of interest were pooled and dialyzed overnight against Dulbecco’s phosphate-buffered saline (PBS) (Sigma-Aldrich) supplemented with 200 mM NaCl at 4°C using SnakeSkin dialysis tubing with a 10 kDa MWCO (Thermo Fisher Scientific). Protein samples were then further concentrated using a 10 kDa Amicon Ultra centrifugal filter (Merck Millipore).

### 4.4 SDS-PAGE

For reducing or nonreducing SDS-PAGE a total of 5 μg of purified protein was loaded on a 4–15% Mini-PROTEAN TGX gel (Bio-Rad Laboratories, United States) and visualized with Coomassie Brilliant Blue staining.

### 4.5 Small-angle X-ray scattering and multi-angle light scattering data acquisition in line with SEC (SEC-MALS-SAXS)

For SEC-MALS-SAXS experiments, 90 µL of samples containing SEC-purified monomeric IgA2 (m2) at circa 40 µM were prepared in PBS pH 7.4 buffer. SEC-MALS-SAXS was collected at the SIBLYS beamline (BL 12.3.1) at the Advanced Light Source (ALS) at Lawrence Berkeley National Laboratory (LBNL) in Berkeley, California (Hura et al., 2009; Classen et al., 2013; Rosenberg et al., 2022). X-ray wavelength was set at λ = 1.27 A, and the sample to detector distance was 2,100 mm, resulting in scattering vectors, q, ranging from 0.01 A^−1^ to 0.45 A^−1^. The scattering vector is defined as q = 4πsinθ/λ, where 2θ is the scattering angle. All experiments were performed at 20°C, and data were processed as described before (Rosenberg et al., 2022). Briefly, a SAXS flow cell was directly coupled with an online Agilent 1260 Infinity HPLC system using a Shodex KW 803 column. The column was equilibrated with running buffer (PBS pH 7.4) with a 0.5 mL/min flow rate. 90 μL of each sample was run through the SEC, and 3 s X-ray exposures were collected continuously during a 25 min elution. The SAXS frames recorded before the protein elution peak were used to subtract all other frames. The subtracted frames were investigated by the radius of gyration (*R*_g_) derived by the Guinier approximation I (q) = I (0) exp (−q2*R_g_*2/3) with the limits q* *R*_g_ <1.5. The elution peak was mapped by comparing integral ratios to background and *R*_g_ relative to the recorded frame using the program BIOXTAS RAW, Supplementary Figure S6 (Hopkins et al., 2017). Final merged SAXS profiles, derived by integrating multiple frames across the elution peak, were used for further analysis, including a Guinier plot, which determined the aggregation-free state. The program GNOM was used to compute the pair distribution function P(r) (Svergun, 1992). P(r) functions for different glycoforms of IgA2, were normalized at the maxima; the area under P(r) function correlates to molecular weight estimated by SAXS. MALS experiments were performed using an 18-angle DAWN HELEOS II light scattering detector connected in tandem to an Optilab refractive index concentration detector (Wyatt Technology). System normalization and calibration were performed with bovine serum albumin using a 45 μL sample at 10 mg/mL in the same SEC running buffer and a dn/dc value of 0.19. UV, MALS, and differential refractive index data were analysed using Wyatt Astra 8 software to monitor the sample’s homogeneity across the elution peak complementary to the SEC-SAXS mentioned above signal validation.

### 4.6 Solution state modeling

Rigid body modeling along with a FoXS and MultiFOXS was used to define, select, and weight the two-state atomistic model that best agreed with SAXS profiles of IgA2 (m2) variants (Schneidman-Duhovny et al., 2016; Schneidman-Duhovny and Hammel, 2018). The initial structural model of IgA2 was predicted with AlphaFold-Multimer. The glycan moieties were built using the glycan reader component of CHARMM-GUI (Jo et al., 2008). Based on mass spectrometry data, complex glycans were built on the sites NVT, NSS and NIT, mannose type glycans on NLT and a shorter glycan was added to the site NVS to resemble the low occupancy (Jo et al., 2008). In the context of glycoproteins, it is recognized that the scattering attributed to glycans exceeds that originating from the protein component alone (Hammel et al., 2002). Minimal molecular dynamics (BILBOMD) simulation applied on the antibody hinge regions (amino acids 228–235), on the tails (amino acids 449–466), and on the glycans explores the Fab domain’s conformational space relative to the Fc-glycan region. BILBOMD performs minimal molecular dynamics (MD) simulations on the flexible regions at very high temperature, where the additional kinetic energy prevents the Fabs from becoming trapped in a local minimum. In the conformational sampling, individual Fab’s would move independently of one another. A single best-fit and two-state models were selected for each IgA2 using MultiFOXS, Supplementary Figure S7 (Schneidman-Duhovny et al., 2016). We estimated the number of states in the solution by examining the *R*_g_ distribution for the top 300 multistate models that all gave the same goodness-of-fit (χ2 ∼ 0.95). The number of main peaks in the distribution indicates the number of states. The area and the position of the peaks validate the level of Fab’s mobility. UCSF Chimera software was used for visualization of PDBs (Pettersen et al., 2004).

### 4.7 SPR spectroscopy

Binding experiments of SEC-purified monomeric IgA glycoforms (MW = ∼150 kDa) to FcαRI (MW = ∼27 kDa) were performed using a Biacore T200 system (Cytiva). All assays were performed with PBS running buffer (Cytiva) at 25°C. All antibodies were coupled to a CM5 Sensor Chip (Cytiva) via amine coupling. All sample dilutions were prepared in PBS supplemented with 0.05% Tween, and 0.1% BSA. Capture of the different IgA variants on the chip surface was performed for 60 s with a concentration of 33 µM and a flow rate of 10 μL/min in acetate buffer pH 5.0. The reference flow cell remained unmodified to serve as a reference cell for the subtraction of systematic instrument noise and drift. FcαRI binding curves were generated in single-cycle kinetic experiments at five different concentrations ranging from 12.5 to 400 nM with 60-s association and 60-s dissociation time at a flow rate of 10 μL/min. After each run, surface regeneration was accomplished using 10 mM glycine, pH 1.7, for 120 s at a flow rate of 30 mL/min. Prior to SPR measurements protein concentration for each sample was verified by averaging 3 independent measurements taken with a NanoDrop spectrophotometer in the UV-mode (at 280 nm using sample-specific extinction coefficients). Binding affinities (K_D_) were calculated with BIAevaluation software using a 1:1 binding model. Measurements were repeated three times and are reported with standard deviation.

### 4.8 *N*-glycan analysis

A total of 20 µg of monomeric purified protein was reduced, S-alkylated, and digested with trypsin (Promega). If required, samples were additionally digested with the endoproteinase Asp-N (Sigma-Aldrich). Glycopeptides were then analysed by capillary reversed-phase chromatography and electron-spray MS using a Bruker Maxis 4G Q-TOF instrument as described previously (Grünwald-Gruber et al., 2017). Site-specific glycosylation occupancy was calculated using the ratio of deamidated to unmodified peptide determined upon *N*-glycan release with peptide:*N*-glycosidase A (Europa Bioproducts).

### 4.9 DSF

Protein thermal shift assays was performed using the FRET channel of a CFX Real-Time PCR Systems (Bio-Rad). IgA2 (m2) glycoforms were diluted in 20 μL at 1 mg/mL in PBS buffer with a final dilution of 1:500 of the Sypro Orange dye stock (Molecular Probes, United States). Melting curves were constructed from 15°C to 90°C (0.5 increments) and data were analysed with the CFX Maestro Software (Bio-Rad). The melting temperatures based on the peak of the first derivative of the fluorescence versus temperature plots were obtained from the software.

### 4.10 CD

CD spectroscopy was performed using a Chirascan™ CD spectrometer (Applied Photophysics, United Kingdom). The instrument was flushed with a nitrogen flow of 5 L/min. The different monomeric IgA2 glycoforms were brought to an absorbance at 280 nm of 0.8 in PBS, pH 7.4. Samples were measured in a cuvette with a path length of 1 mm in the far-UV region ranging from 190 to 260 nm, a 5-nm/s scan speed, and a 3-nm bandwidth. The transition from folded state to unfolded state is monitored by measuring the ellipticity values at 222 nm upon gradually heating from 20°C to 90°C, with heating rates 0.5°C/min.

### 4.11 nanoDSF

NanoDSF was performed using Prometheus equipped with back reflection mode (NanoTemper Technologies, München, Germany). SEC-purified monomeric IgA2 (m2) were loaded in nanoDSF grade standard capillaries and exposed at temperatures from 15°C to 90°C using a thermal ramping rate of 1°C/min. Fluorescence emission from tryptophan after UV excitation at 280 nm was collected at 330 nm and 350 nm with a dual-UV detector. Thermal stability parameters were calculated using the ThermControl software (NanoTemper Technologies, München, Germany).

## Data Availability

The raw data supporting the conclusion of this article will be made available by the authors, without undue reservation.
