# Supplementary material for: Effects of N-glycans on the structure of human IgA2
Source: Front Mol Biosci. 2024 Apr 5;11:1390659. doi: 10.3389/fmolb.2024.1390659 (PMC11026580; doi:10.3389/fmolb.2024.1390659)
Supplement: Supplementary file 1 [file DataSheet1.PDF]

Supplementary Material

Effects of *N*-glycans on the structure of human IgA2

Valentina Ruocco[1], Clemens Grünwald-Gruber[2], Behzad Rad[3], Rupert Tscheliessnig[4], Michal Hammel[5], Richard Strasser[1*]

[1]Department of Applied Genetics and Cell Biology, University of Natural Resources and Life Sciences, Vienna, Austria

[2]Core Facility Mass Spectrometry, University of Natural Resources and Life Sciences, Vienna, Austria

[3]The Molecular Foundry, Lawrence Berkeley National Laboratory, 94720, CA, USA

[4]Division of Biophysics, Gottfried-Schatz-Research-Center, Medical University of Graz, Austria

[5]Molecular Biophysics and Integrated Bioimaging, Lawrence Berkeley National Laboratory, Berkeley, 94720, CA, USA

*Correspondence:

Richard Strasser

Email: richard.strasser@boku.ac.at

Keywords: *N*-linked glycan, IgA antibodies, SAXS, flexibility, protein assembly, protein stability

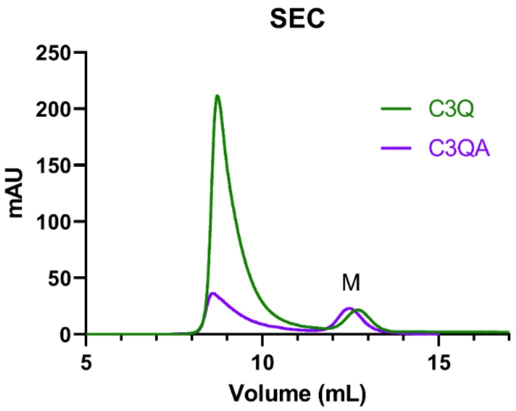

Supplementary Figure 1. Size exclusion chromatography profiles of the C3Q and C3QA variants.

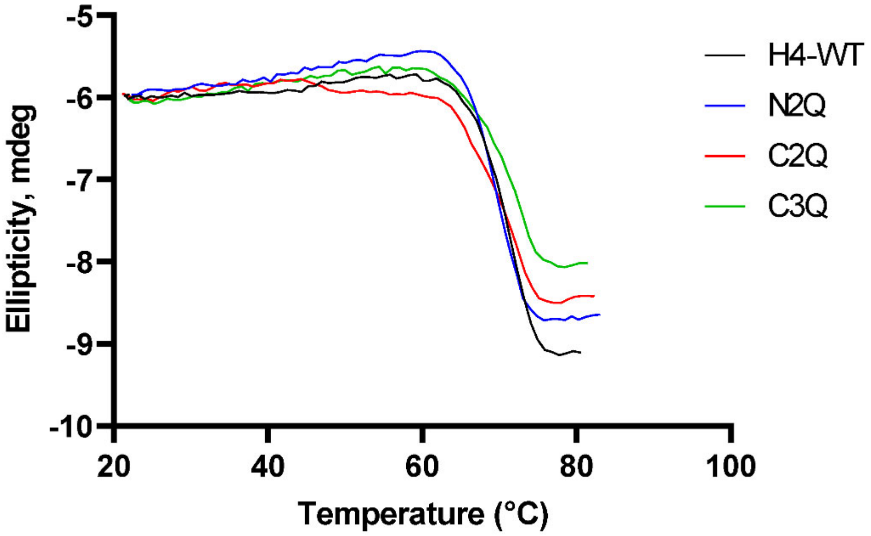

Supplementary Figure 2. Thermal denaturation curves of IgA2(m2) variants at 222 nm in PBS buffer. Heating rate 0.5°C/min.

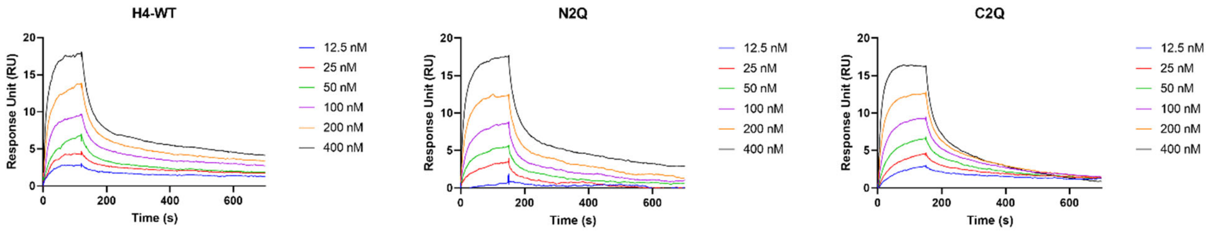

Supplementary Figure 3. SPR sensorgrams showing the binding of IgA2m2 to FcαRI. Experiments were done in three runs using concentrations from 400nM to 12.5nM; one representative run is shown. A running buffer (blank) was applied before and after each experiment and was subtracted from the binding response.

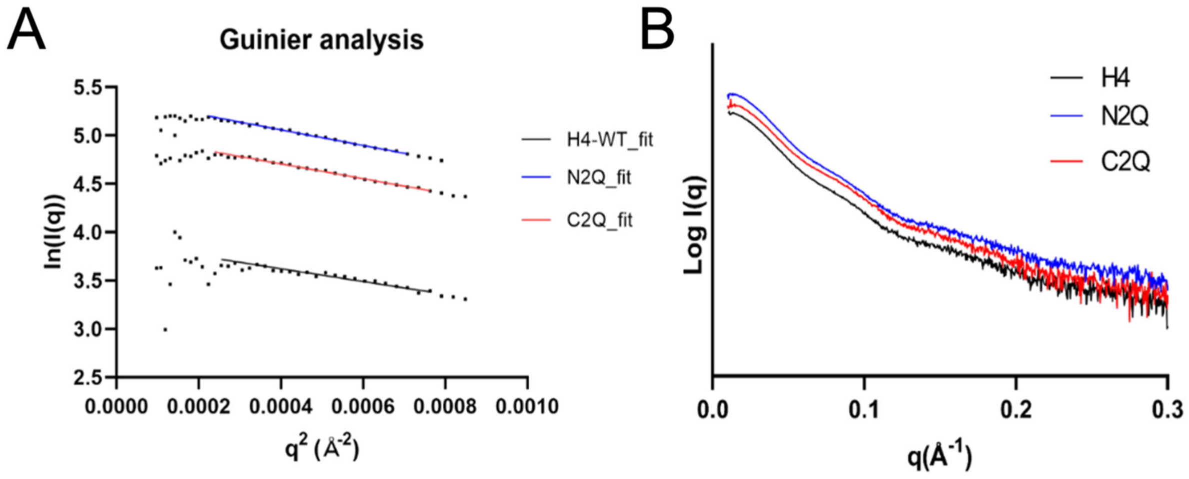

Supplementary Figure 4. (A) Guinier analysis of the IgA2(m2) variants. (B) Experimental SAXS curves.

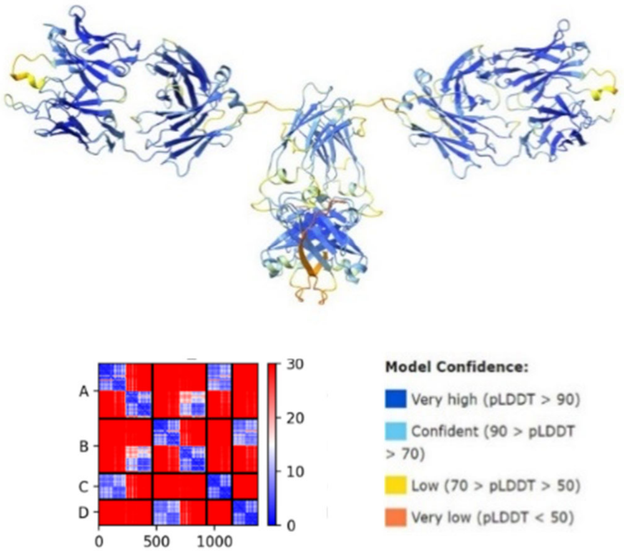

Supplementary Figure 5. Cartoon model of the structure of IgA2m2 showing the pLDDT per position, which is integrated as the b-factor in the PDB file delivered by Alphafold2 in rainbow colors ranging from red (high confidence) to blue (low confidence). (C) Prediction aligned error (PAE) score for model ranked 1. This score displays the calculated error of the predicted distance for each pair of residues. Both axes indicate the position of the individual amino acids. The uncertainty in the predicted distance of two amino acids is color coded from blue (0 Å) to red (30 Å), as shown in the right bar. The color of the intersection of a horizontal line drawn from the position of an amino acid on the y-axis and a vertical line from the position of another amino acid on the x-axis indicates the error in the predicted distance between these two residues. PAE graphs are characterized by a diagonal blue line, since amino acids that are juxtaposed in the primary sequence are also adjacent in the 3D structure.

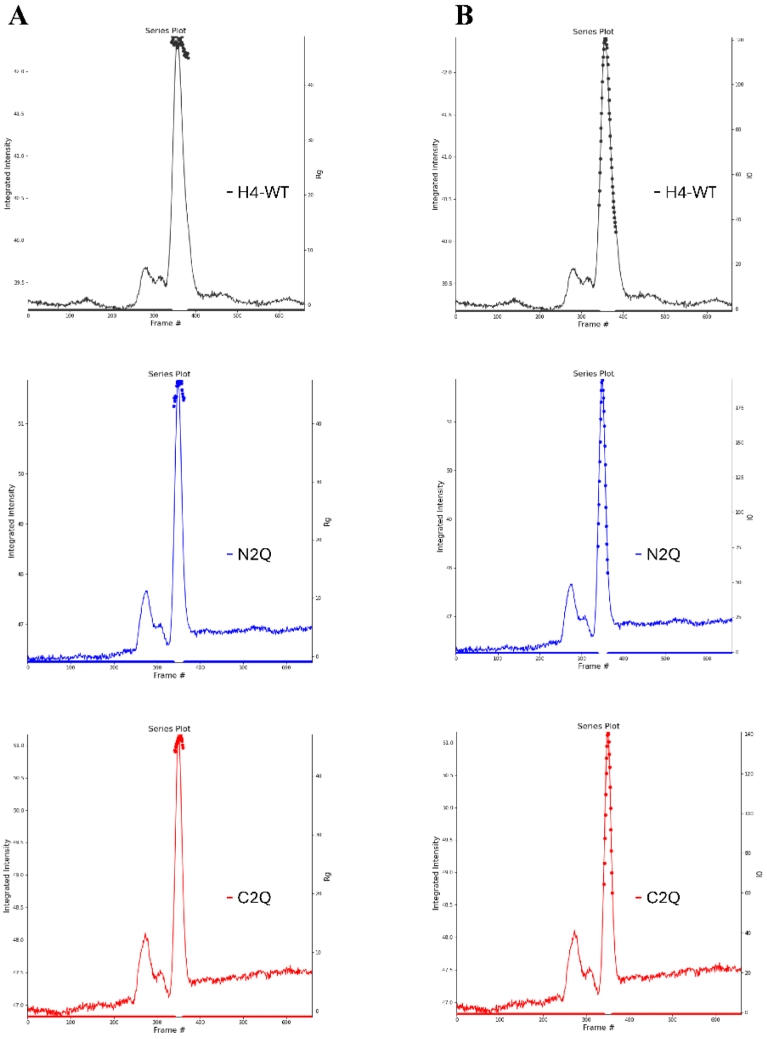

Supplementary Figure 6. Buffer subtracted SAXS chromatographs for the IgA2(m2) variants using BioXTAS RAW. R_g (A) and I(0) (B) are calculated in the frames #351-363 for H4-WT, #336-368 N2Q and #334-371 for C2Q.

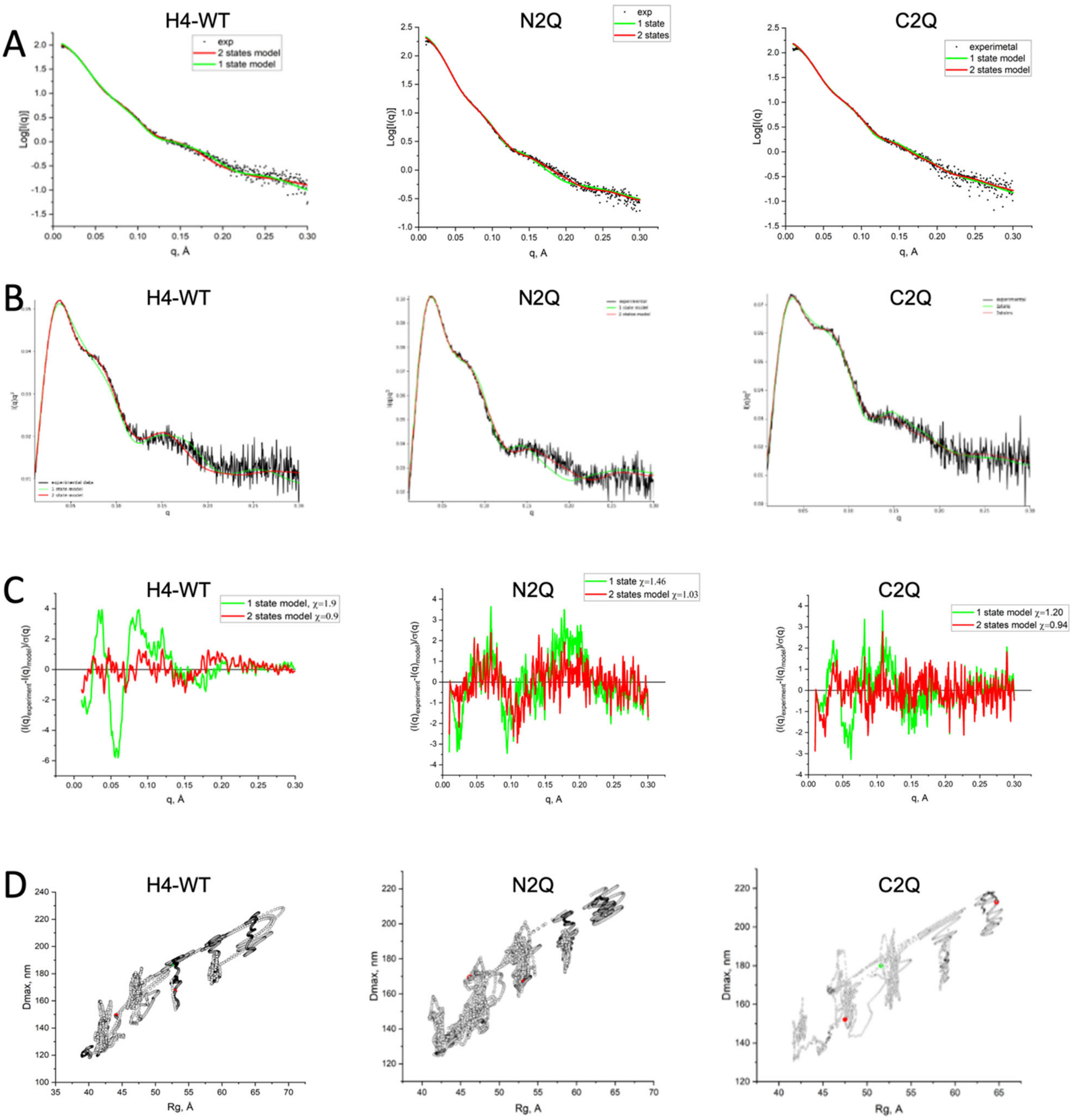

Supplementary Figure 7. Modeling parameters and results. (A) Experimental SAXS profiles (dot) and theoretical SAXS profiles calculated from their respective one-state (green) and two-state atomistic models (red). (B) Experimental Kratky plot profiles (black) and theoretical Kratky profiles calculated from their respective one-state (green) and two-state atomistic models (red). (C) Residuals (experiment/model) for the fits of one state and two-state models. (D) The distribution of R_g and Dmax was explored during the simulation with BILBOMD.
